# The association between objectively measured physical activity and health-related quality of life, life-space mobility and successful ageing in older Indian adults

**DOI:** 10.4102/hsag.v27i0.1638

**Published:** 2022-01-28

**Authors:** Jeanne M. Grace, Jacqueline Naiker

**Affiliations:** 1 Department of Biokinetics, Exercise and Leisure Sciences, College of Health Sciences, University of Kwazulu-Natal, Durban, South Africa

**Keywords:** active life expectancy, geriatrics, nursing homes, retirement, well-being

## Abstract

**Background:**

Longevity is increasing, accompanied by a rise in disability and chronic diseases with physical activity (PA) delaying disability, ensuring successful ageing (SA) and independent living in older adults.

**Aim:**

This study aimed to determine objectively measure PA levels, health-related quality of life (HRQoL), life-space mobility and SA of older adults as well as their mutual associations.

**Setting:**

KwaZulu-Natal province, South Africa.

**Methods:**

A total of 210 older adults aged 65–92 years were purposively sampled and completed the Medical Outcomes Study 36-Item Short-Form Health Survey, the Life-Space Mobility, and Successful Ageing questionnaires. Physical activity levels were measured using an Omron Pedometer, which the participants wore for seven consecutive days.

**Results:**

The average number of steps taken per day for the 7 days was 2025, with 98.6% of the entire study population classified as sedentary. The Vitality domain (one of 8 categorised) reflected the best health status (M = 59.9, s.d. ± 18.8) with a significant 93% of the participants indicating that they had not visited places outside their immediate neighbourhood (*p* < 0.0005). A significant, negative association between the average number of steps taken in 7 days and all three SA variables, namely, the physical (*r* = –0.152, *p* = 0.027), sociological (*r* = –0.148, *p* = 0.032) and psychological (*r* = –0.176, *p* = 0.010), and a significant, positive association with life-space mobility (*r* = 0.224, *p* = 0.001) was noted.

**Conclusion:**

The majority of the older adults were sedentary, affecting their HRQoL, life-space mobility, and SA negatively.

**Contribution:**

It is imperative to develop effective physical activity programmes to ensure successful ageing by improving older adults’ quality of life and physical activity levels.

## Introduction

The biggest threat to healthy ageing is sedentary living, with the ‘golden years’ of most individuals affected by health conditions exacerbated by unhealthy lifestyles (Active Living Coalition for Older Adults 2018; Naiker 2018). Hence, helping people to age better, delay functional decline, and reduce the risk of premature mortality is an essential objective for health professionals, which can be achieved when older adults participate in regular physical activity. The Active Living Coalition for Older Adults (2011) emphasises that being physically active is one of the best lifestyle choices that can enhance an individual’s health and enable a longer and healthier life. Also, the Aged Care and Housing Group (2018) states that no matter an individual’s age, one is never too old to participate in physical activity – which can be adjusted to accommodate a person’s abilities and health status.

Several studies have shown that step count interventions successfully increase habitual physical activity in older adults (Hobbs et al. 2013; Ueno et al. 2013). A goal of 10 000 steps per day is often cited as a recommended target to maintain good health (Tudor-Locke et al. 2011). By translating 150 min of moderate-intensity physical activity (MVPA) into steps taken per day, healthy older adults are recommended to accomplish at least 7100 steps per day when averaged over a week. In the case of older adults with chronic conditions or disabilities, a minimum number of 4600 steps per day is recommended, if averaged over a week (Tudor-Locke et al. 2011). An activity level of less than 5000 steps per day for an older adult is generally considered to be too low and regarded as an indicator of a ‘sedentary lifestyle’ associated with a poor health-related quality of life (HRQoL) and potentially leading to an elevated incidence of cardiovascular risk factors, obesity, and depression (Tudor-Locke et al. 2013). Of concern is that only 20% – 60% of older adults globally meet the physical activity recommendations, depending on the assessment method used (Aoyagi & Shephard 2013; Sun, Norman & While 2013). Most studies conducted on healthy older adults, a markedly heterogeneous population, span a wide normative step range of 2000–9000 per day (Tudor-Locke et al. 2011); with none having recorded step data reflecting the physical activity levels of older South African adults.

Researchers assert that the primary goal of health-enhancing physical activity is to improve and preserve HRQoL (Olivares et al. 2011), with Ludendorff Queiroz et al. (2016) and Bogen et al. (2017) establishing associations between physical activity and HRQoL, because physical activity greatly influences the HRQoL of older adults. Findings on the influence of physical activity on different HRQoL domains vary, with most studies relying on the subjective assessment of physical activity, whereas using objective methods has been reported in only a limited number of studies. The relationship between the physical activity levels of older South African adults and their HRQoL, for example, remains to be established.

Successful ageing (SA), meaning how satisfactorily older people age physically, psychologically and socially, is affected by sedentary behaviour (Dogra & Stathokostas 2012). Researchers reported that physically active older individuals were more than twice as likely to be rated as ageing successfully compared to those who neglected such activity (Baker, Bodner & Allman 2003). Ageing is marked by impaired health, age-related diseases, a decrease in physical condition and activity, reduced physical attractiveness, loneliness, disability, as well as reliance on and difficulties accessing adequate healthcare and nursing services (Choi et al. 2017). Older adults must, therefore, develop skills and behaviours, such as becoming physically active, that can help them to age gracefully (Dogra & Stathokostas 2012). However, whether older South African adults age successfully and benefit from physical activity in this regard is unknown.

For older adults, mobility not only plays a vital role in maintaining an independent lifestyle but also preserves and maintains their physical and psychological health, as well as total well-being (Portegijs et al. 2014; Rantakokko, Mänty & Rantanen 2013). Hence, it is essential to obtain insight into their movement through different life-space areas such as bedroom, home, yard, neighbourhood, town and beyond in their daily activity as it can reflect early signs of mobility decline. In this regard, some researchers have found that older people with difficulty moving independently beyond their neighbourhood, might be susceptible to an inactive lifestyle confirmed by a dependence of daily step counts on life-space mobility (Tsai et al. 2015). Consequently, it is essential to determine the life-space mobility of older adults in South Africa and its association with their objectively measured physical activity levels – information that is currently not known.

It is within the broad context described above that this study primarily aims to determine the possible effect of the physical activity levels of older adults in KwaZulu-Natal, a province in South Africa, on their HRQoL, SA, and life-space mobility. A secondary aim was to establish, for the first time in this setting, their objectively measured physical activity levels, HRQoL, SA and life-space mobility. Obtaining such information in the South African context is crucial to the policies of relevant state authorities, nursing homes, gerontologists and health professionals to understand better the role and consequences of physical inactivity in older adults and propose interventions to improve the course of their ageing.

## Methods

### Setting, population and sample

This cross-sectional study involved 210 males and females aged 65–92 years (M = 72.2, s.d. = 6.1), recruited from a population of 375 residing in two nursing homes that were conveniently sampled in a suburb of Durban, KwaZulu-Natal. To provide a statistical power of 80% and a significance level of 95%, for a population of 375, a sample of 210 men and women was purposively sampled with inclusion criteria of their being older than 65 years, able to proceed without a walking device, with no orthopaedic limitation. Potential participants did not qualify if they were unable to walk because of ill-health or disability or had mental health (MH) problems and limited writing ability.

### Interventions

#### Objectively measured physical activity levels

Physical activity was measured using an Omron Pedometer Walking Style Pro 2.0 instrument (OMRON, Kyoto, Japan), worn by the study participants for seven consecutive days following the completion of various questionnaires. The pedometer was given with detailed instructions provided orally and on paper. The participants were instructed to wear the pedometer on the right hip during waking hours except when taking a sauna, bathing, or engaging in other activities exposed to water. They were encouraged to maintain their usual daily routines during the measurement period. The study population was given an activity diary in which they recorded the times they put on and took off the pedometer during the 7 days. After a week, the instrument was returned to the researcher. When the accompanying diary reported at least 10 h of pedometer wear time, the day in question was considered valid for evaluation. Data on any participant for less than four valid days or more than 1 day between consecutive diary dates were excluded.

#### Health-related quality of life

Health-related quality of life was assessed using the Medical Outcomes Study Short-form Health Survey (SF-36) (Hays, Sherbourne & Mazel 1993). This 36-item measure is organised into eight domains, namely physical functioning (PF), role physical health (RPH), bodily pain (BP), general health (GH), vitality (VT), social functioning (SF), role emotional health (REH) and MH. The eight domains were scored from 0 to 100, indicating the worst and best possible health status measures.

#### Successful ageing

Successful ageing variables were created for all three components of this metric, namely, physical (presence of chronic disease and functional impairments), psychological (cognitive function, emotional vitality, and depression), and sociological (engagement with life, social support, and spirituality). Researchers recently outlined the required variables to assess each of the components of SA based on Rowe and Kahn’s model (Rowe & Kahn 1987; Young, Frick & Phelan 2009). Each SA component for the current analysis was based on the outline of Young et al. (2009).

#### Life-space mobility

Life-space mobility was measured with the Life-Space Assessment questionnaire (Baker et al. 2003), which collects information about mobility habits in a continuum of five environments or life-space levels: within the home, around the house, in the neighbourhood, in town, and outside of town. Our study population was asked if they had attained any of the five life-space levels over the last 3 days, at what frequency, and whether assistive devices were used or human assistance was needed. From 20 items, a composite score ranging from 0 to 120 was computed. As the questionnaires were tested in a pilot study, the last three items were excluded from the final questionnaire as they were not deemed appropriate to the current sample.

The three questionnaires were self-administered at the study centre by those participants capable of doing so independently. The research coordinator administered the questionnaires to participants who were unable to complete the surveys on their own.

### Data analysis

Descriptive statistics of the participants’ particulars and their physical activity levels were reported as means and standard deviations. The frequency distribution of responses from the SF-36 and the Life-space mobility questionnaires are presented in tables. A z-test approximation of the binomial test was used to test whether a proportion of the questions answered yes or no to the Life-space mobility questionnaire was significant. For SA, the Kaiser–Meyer–Olkin Measure of Sampling Adequacy and Bartlett’s Test of Sphericity were used to determine the data’s suitability for structure detection. Internal consistency reliability was determined using Cronbach’s alpha coefficient (α) for all scales of the three SA variables and the eight SF-36 health domains. A one-sample *t*-test was applied to test for significance of agreement or disagreement for each of the three SA variables. Pearson product-moment correlation coefficient was used to determine the association of physical activity with HRQoL, life-space mobility and SA. Throughout, a *p*-value of 0.05 was used to indicate significance. The analysis was carried out using SPSS (Statistical Package for Social Sciences) version 23 (IBM Corp Armonk, NY).

### Ethical considerations

All procedures performed in this study involving human participants were in accordance with the World Medical Association (2013) and its later amendments or comparable ethical standards. Ethical clearance was obtained from the University of KwaZulu-Natal’s Biomedical Research and Ethics Committee (BFC609-17). Written informed consent was obtained from the participants, and their anonymity was preserved.

## Results

### Demographics and physical activity levels

Two hundred and ten older adults participated in the study, ranging between 65 and 92 (M = 72.2, s.d. = 6.09) years, with 68% of the sample between the ages of 65 and 79. The majority of the participants were women (62%, *n* = 130), with 92% (*n* = 194) being of Indian ethnicity. The minimum and maximum of the total number of steps taken by the participants in 7 days were 1386 and 66 815 (M = 14 074, s.d. = 8644), respectively. The average number of steps taken per day for the 7 days was 2025 (s.d. = 1352), with a minimum and maximum of 198 and 9549, respectively.

### Health-related quality of life score

The scores for the eight domains were M = 41.1 (± 23.8; α = 0.92) (PF), M = 55.7 (± 46.4; α = 0.95) (RPH), M = 53.1 (± 19.0; α = 0.59) (BP), M = 56.3 (± 19.6; α = 0.55 (GH), M = 41.0 (± 43.9; α = 0.88) (REH), M = 59.9 (± 18.8; α = 0.56) (V), M = 43.5 (± 19.7; α = 0.75) (MH), and M = 53.6 (± 20.6; α = 0.53) (SF), respectively. The means, SDs and Cronbach’s alpha values for the eight subscales of the HRQoL scores are presented in Table 1. The higher the mean score for a variable, the better the participant’s health status in that respect, hence, the Vitality domain reflects the best health status (M = 59.9, s.d. ± 18.8; α = 0.56), whereas the REH domain represents the poorest health status (M = 41.0, s.d. = ± 43.9; α = 0.75).

**TABLE 1:** Health-related quality of life scores of eight domains measured by SF-36.

Scale	M	± s.d.	Alpha (α)
Physical functioning (PF)	41.1	23.8	0.92
Role physical health (RPH)	55.7	46.4	0.95
Bodily pain (BP)	53.1	19.0	0.59
General health (GH)	56.3	19.6	0.55
Role emotional health (REH)	41.0	43.9	0.88
Vitality (V)	59.9	18.8	0.56
Mental health (MH)	43.5	19.7	0.75
Social functioning (SF)	53.6	20.6	0.53

s.d., standard deviation.

### Successful ageing

All three variable scores (physical, sociological and psychological) of the SA scale for the study sample was statistically different from the normed value of 4 (Table 2). Cronbach’s alpha for the physical, sociological and psychological variables presented in Table 2 was 0.77, 0.94, and 0.95, respectively. The results show that the older adults were successfully coping in respect of all three SA variables.

**TABLE 2:** Results of one-sample *t*-test and Cronbach’s alpha for successful ageing variables (*n* = 210).

SA variable	Mean	s.d.	Test value	Mean difference	95% CI of difference		*t*	*df*	Alpha
					Lower	Upper			
Physical	1.66	0.75	4	−2.34	−2.44	−2.24	−45.37*	209	0.77
Sociological	1.36	0.84	4	−2.64	−2.76	−2.53	−45.71*	209	0.94
Psychological	1.83	1.13	4	−2.17	−2.33	−2.02	−27.64*	209	0.85

SA, successful ageing; s.d., standard deviation; CI, confidence interval; *t, t* score; *df*, degrees of freedom.

* , *p* < 0.0005.

### Life-space mobility

Table 3 demonstrates that a significant 196 (93%) participants of the study sample reported that during the previous 3 days, they had been to other rooms of their home beside the room where they sleep (*p* < 0.0005). A significant 190 (90%) reported that they had been to an area immediately outside their homes, such as the porch, deck or patio, hallway of an apartment building, or garage (*p* < 0.0005). A significant 137 (65%) indicated that during the previous 3 days, they had been to an area outside their home, such as a yard, courtyard, driveway or parking lot (*p* < 0.0005). A significant 196 (93%) and 139 (66%), respectively, declared that they had not visited places outside their immediate neighbourhood that are neither within their town or community nor beyond their immediate town or community (*p* < 0.0005).

**TABLE 3:** Responses from life-space mobility questionnaire.

Questions	Response	*n*	%	*p*
1. During the past 3 days, have you been to other rooms of your home besides the room where you sleep?	-	-	-	< 0.0005†
	Yes	196	92.9	-
	No	15	7.1	-
2. During the past 3 days, have you been to an area immediately outside your home such as your porch, deck or patio, the hallway of an apartment building, garage?	-	-	-	< 0.0005†
	Yes	190	90	-
	No	21	10	-
3. During the past 3 days, have you been to an area outside your home such as a yard, courtyard, driveway, or parking lot?	-	-	-	< 0.0005†
	Yes	137	64.9	-
	No	74	35.1	-
4. During the past 3 days, have you been to places in your immediate neighbourhood, but beyond your own property or apartment building?	-	-	-	0.7830
	Yes	103	48.8	-
	No	108	51.2	-
5. During the past 3 days, have you been to places outside your immediate neighbourhood but within your town or community?	-	-	-	< 0.0005†
	Yes	15	7.1	-
	No	196	92.9	-
6. During the past 3 days, have you been to places outside your immediate town or community?	-	-	-	< 0.0005†
	Yes	72	34.1	-
	No	139	65.9	-

† , Based on *Z* approximation.

### Association between average number of steps taken in 7 days and health-related quality of life

There were no significant correlations between the average number of steps taken by the study population over 7 days and their HRQoL (Table 4).

**TABLE 4:** Association between average number of steps and health-related quality of life.

Average number of steps in 7 days	Standard deviation	HRQoL domains	*r*	*p*
2025	± 1352	Physical functioning	−0.059	0.395
		Role physical health	−0.112	0.107
		Bodily pain	−0.053	0.444
		General health	−0.015	0.825
		Role emotional health	−0.065	0.325
		Vitality	−0.038	0.587
		Mental health	−0.066	0.344
		Social functioning	−0.024	0.401

HRQoL, health-related quality of life; *r*, correlation coefficient.

### Association between average number of steps in 7 days and successful ageing

Table 5 displays a negative, weak but significant correlation between all three SA variables, namely physical (*r* = –0.152, *p* = 0.027), sociological (*r* = –0.148, *p* = 0.032), psychological (*r* = –0.176, *p* = 0.010), and the average number of steps taken in 7 days.

**TABLE 5:** Association between average number of steps and life-space mobility.

Average number of steps in 7 days	Standard deviation	SA variables	*r*	*p*
2.025	± 1.352	Physical	−0.152†	0.027
		Sociological	−0.148†	0.032
		Psychological	−0.176†	0.010

SA, successful ageing; *r*, correlation coefficient.

† , Correlation is significant at the 0.05 level (2-tailed).

### Association between average number of steps in 7 days and life-space mobility

There was a positive, weak correlation between the average number of steps taken in 7 days (2025 ± 1352) and life-space mobility (*r* = 0.224, *p* = 0.001).

## Discussion

This is the first study to report on the association of the objectively measured physical activity levels of older men and women in South Africa regarding their HRQoL, SA, and life-space mobility. Of concern is that only 0.9% of the study population met researchers’ proposed recommended 7100 steps per day that translate 150 min of MVPA into steps per day for healthy, older adults (Naiker 2018; Tudor-Locke et al. 2011). The 0.9% compares poorly with the findings of researchers who reported that 20% – 60% of older adults globally met the minimum steps requirement (Aoyagi & Shepard 2013; Sun, Norman & While 2013). Also, 98.6% of the survey population are regarded as sedentary with an activity level of less than 5000 steps per day, which is associated with a relatively high prevalence of cardiovascular risk factors and poor HRQoL (Naiker 2018; Tudor-Locke et al. 2013). There were no significant correlations between the 2025 average number of steps taken in 7 days by the participants and their HRQoL. Dohrn reported a moderate to strong association between physical activity and HRQoL with the former’s influence varying in the different domains (Dohrn 2015; Naiker 2018). The fact that the Vitality domain reflected the best health status, with a mean score of 59.9, could be explained by the participants’ meagre average step count. Besides, the physical function domain reflected the second-poorest health status (41.1), which corresponds with other researchers’ findings who concluded that seniors’ daily life walking steps per day was positively associated with health-related PF (Bogen et al. 2017; Naiker 2018).

Our results also showed that the older men and women were successfully coping in all three SA (physical, social and psychological) variables, in contrast to Dogra and Stathokostas’ (2012) findings. However, our conclusion that there was a negative, weak and significant correlation between the average number of steps taken per day and the three SA variables were expected based on research about physical activity and SA (Naiker 2018). Researchers noted a direct association between these factors (Baker et al. 2009); other researchers reported that physical activity strongly relates to SA and each of its components, such that higher levels of physical inactivity were associated with an increased likelihood of reporting disease and disablement, low functional capacities, and being socially disengaged with life (Choi et al. 2017; Dogra & Stathokostas 2012). It is important to note that the older adults’ physical activity levels in the research studies referred to above were subjectively measured compared to our study, which used an objective means of determining the participants’ physical activity levels.

The older adults’ life-space mobility in our study correlated positively with their average number of steps taken in 7 days, which is corroborated by the findings reported in other studies (Aoyagi & Shephard 2013; Naiker 2018; Tsai et al. 2015). However, upon the investigation of their life-space mobility areas, it is interesting to note that a significant number of these people’s mobility habits took place in three of the five life-space environments, namely, outside their room, immediately outside and around their home, but not outside their immediate neighbourhood or beyond their immediate town or community. A plausible explanation for the above finding, also reflected in their low average number of daily steps, is that older adults are dependent on transport to enable them to move into their immediate neighbourhood and beyond. In support of this finding, Portegijs et al. (2014) showed that a sense of autonomy in outdoor activities explained a substantial part of the variation in life-space mobility in healthy older people, thereby indicating that physical and psychosocial factors play a role in maintaining mobility in old age.

## Conclusion

This research has added to the body of knowledge on older adults’ physical activity levels in South Africa with the novelty of associating their physical activity with their HRQoL, SA, and life-space mobility. The most important contribution is that 98.6% of the study sample was sedentary, which affected their HRQoL, SA and life-space mobility adversely. The above is underscored by the results that showed no significant correlation between the average number of steps taken and their HRQoL. Moreover, the relationship between their average number of steps and SA was negative. Their unsatisfactory life-space mobility further demonstrates this as these men and women are not moving outside their immediate neighbourhood, town, or community. With the dose-response relationship between physical activity and health well established, the participants’ low physical activity levels in this study and their consequences are of concern when considering that an increased number of active and financially stable older adults live well into their 90s. Hence, it has become imperative to encourage SA by such means as improving their quality of life and physical activity levels. Thus, it is essential not only to develop effective physical activity programmes but also to convince state authorities and nursing home organisations to implement and support such programmes.

## Limitations

Chronic medical diseases, for example, diabetes, dyslipidaemia, hypertension, angina, osteoporosis, and arthritis of the older adults in the present study, were not recorded. Some researchers recommend that a minimum step count of 4600 steps per day, if averaged over a week, is desirable to include 150 min of MVPA for older adults with chronic diseases (Tudor-Locke et al. 2011). If the current study population’s chronic diseases were considered, it could possibly better explain their low average step count. The majority of the participants in our study were of Indian ethnicity. Therefore, it is impossible to generalise the results to older people of other races and with chronic medical diseases.
